# Acute cocaine-related health problems in patients presenting to an urban emergency department in Switzerland: a case series

**DOI:** 10.1186/1756-0500-7-173

**Published:** 2014-03-25

**Authors:** Michael Bodmer, Florian Enzler, Evangelia Liakoni, Marcel Bruggisser, Matthias E Liechti

**Affiliations:** 1 Division of Clinical Pharmacology and Toxicology, Department of Biomedicine and Department of Clinical Research, University Hospital Basel and University of Basel, Basel, Switzerland; 2 Department of Internal Medicine, University Hospital Bern, Bern, Switzerland

**Keywords:** Cocaine, Drug abuse, Intoxication

## Abstract

**Background:**

Emergency departments may be a useful information source to describe the demographics and clinical characteristics of patients with acute cocaine-related medical problems. We therefore conducted a retrospective analysis of 165 acute, laboratory-confirmed cocaine intoxications admitted to an urban emergency department in Switzerland between January 2007 and March 2011.

**Results:**

A total of 165 patients with a mean age of 32 years were included. Most patients were male (73%) and unemployed (65%). Only a minority (16%) had abused cocaine alone while 84% of the patients had used at least one additional substance, most commonly ethanol (41%), opioids (38%), or cannabis (36%) as confirmed by their detection in blood samples. The most frequently reported symptoms were chest pain (21%), palpitations (19%), anxiety (36%) and restlessness (36%). Psychiatric symptoms were present in 64%. Hypertension and tachycardia were observed in 53% and 44% of the patients, respectively. Severe poisonings only occurred in patients with multiple substance intoxication (15%). Severe intoxications were non-significantly more frequent with injected drug use compared to nasal, oral, or inhalational drug use. Severe complications included acute myocardial infarction (2 cases), stroke (one case), and seizures (3 cases). Most patients (75%) were discharged home within 24 h after admission. A psychiatric evaluation in the ED was performed in 24% of the patients and 19% were referred to a psychiatric clinic.

**Conclusions:**

Patients with acute cocaine intoxication often used cocaine together with ethanol and opioids and presented with sympathomimetic toxicity and/or psychiatric disorders. Severe acute toxicity was more frequent with multiple substance use. Toxicity was typically short-lasting but psychiatric evaluation and referral was often needed.

## Background

After cannabis, cocaine is the most-used illicit drug in Europe [1-3]. In Europe, 4.3% of adults aged 15–64 have used cocaine at least once in their lifetime [4]. In Spain, as many as 5.9% of people aged 15–64 years reportedly used cocaine at least once during their lifetime [2]. Use of cocaine among Swiss adolescents has increased from 3.8% in 1993 to 9.8% in 2002 [5] and the lifetime prevalence of cocaine use in Swiss adolescents and adults also rose from 1.7% in 2002 to 2.8% in 2007 [6].

Cocaine is one of the leading drugs of abuse resulting in presentations to emergency departments (EDs) in the Western world. Cocaine-related ED visits have increased by 47% in the US from 1995 to 2002 [7]. Cocaine was second only to ethanol as the cause of acute intoxication in patients who presented to a Spanish ED [2].

Cocaine is a central nervous system stimulant which produces euphoria, restlessness, and reduced appetite [8-10]. It is commonly abused by inhalation, nasal insufflation or intravenous injection. Cocaine can affect all body systems potentially resulting in many adverse effects. Among the most severe complications are seizures, hemorrhagic and ischemic strokes, myocardial infarction (MI), aortic dissection, rhabdomyolysis, mesenteric ischemia, acute renal injury, and multiple organ failure [11]. Results from two retrospective ED studies in the US suggested that cocaine-related problems are short-lived in most cases and primarily include cardiovascular (chest pain, palpitations) and neuropsychiatric (dizziness, headache, restlessness, anxiety, psychosis) symptoms [12,13]. Cocaine increases myocardial oxygen consumption and induces coronary artery vasoconstriction [14], facilitates platelet aggregation, and thrombus formation [15-18]. Furthermore, cocaine predisposes to ventricular arrhythmia [19]. Accordingly, acute myocardial infarction (MI) is the most frequent severe cardiovascular complication in cocaine abusers. Three large studies showed that 4-6% of patients who present with cocaine-related chest pain sustain acute MI [20-22]. Ischemic or hemorrhagic stroke, sudden death or seizures related to cocaine use have also been infrequently reported [8,12].

Information regarding cocaine- and other illicit drug-related health problems is scarce in Europe. EDs may be important sources of information about drug-related health consequences [1,23]. Reports from 30 European countries regarding cocaine-related health emergency episodes have recently been summarized for the period from 2007 to 2010 [1]. However, most of these reports concerned calls to poison centers, or admissions to general hospitals, psychiatric hospitals or toxicology clinics. Only 7 reports used EDs as information sources and detailed case descriptions were not presented [1]. The present study aimed to describe in more detail the demographics, clinical findings, management, and short-term outcome of patients with analytically-confirmed acute cocaine use presenting to an urban ED in Switzerland.

## Methods

This observational study was approved by the ethics committee of the cantons of Basel. We retrospectively identified and included all patients admitted to the ED of the University Hospital of Basel with acute cocaine-related medical problems between January 2007 and March 2011. The ED is both a primary care facility (walk-in patients) and a tertiary referral center for hospitals in the greater Basel area. Additionally, all patients brought by the paramedics are first admitted to the ED. All ED presentations are entered into the comprehensive electronic patient chart data-base. We performed full-text search to identify all cases with notions of “cocaine”, or related terms (“freebase” or “crack”) in their electronic chart. The search was performed in the entire text entered by the admitting physician. The entire charts of all cases were then reviewed including all the data obtained by paramedics. Only cases with acute cocaine intoxication were included in the study. A case with acute cocaine-related medical problems (acute cocaine intoxication) was defined as a patient with any medical problem related to self-reported cocaine use within the last 12 h and confirmation of the presence of cocaine in blood serum by the CEDIA cocaine immunoassay (Thermo Fisher Scientific, Bonn, Germany). Patients who did not admit to the use of cocaine were also included in the study if they presented with typical symptoms and signs of sympathetic stimulation and a positive laboratory test for cocaine. Patients with self-reported cocaine use more than 12 h before presentation or patients with a negative blood test as well as body packers and body stuffers were excluded from the study. Analytical confirmation of the use of other drugs of abuse including cannabis, amphetamines including MDMA, benzodiazepines, methadone, opioids, and LSD was also performed using CEDIA immunochemical assays in blood serum. Ethanol blood levels were determined by an enzyme assay. Blood levels of γ-hydroxybutyrate (GHB) levels were determined by high-pressure liquid chromatography only in patients with suspected GHB intoxication. All laboratory analyses were performed in the hospital clinical chemistry department according to good laboratory practice. Data abstracting was performed in a standardized manner by one of the authors for the entire study. Demographic data included age, sex, hour and weekday of ED admission, and employment status. The medical history included the reason for admission, present symptoms, history of drug use, route of drug administration, and context of use. We also recorded any co-ingestion of ethanol or other drugs as reported by the patient and as confirmed in the toxicological blood analyses described above. Clinical variables included the Glasgow Coma Scale (GCS) score, heart rate, blood pressure, respiratory rate, body temperature, oxygen saturation, and laboratory tests. Patients were divided into two groups: patients who used only cocaine and patients who used cocaine together with other drugs based on the blood tests. Severity of poisoning was assessed using the Poison Severity Score for grading acute poisoning [24]. Mild toxicity refers to mild, transient and spontaneously resolving symptoms, moderate toxicity refers to pronounced or prolonged symptoms, and severe toxicity indicates severe or life-threatening symptoms. Differences in the frequency of clinical characteristic between the cocaine monointoxicated and the polyintoxicated patients (according to laboratory findings) were assessed using Fisher’s exact tests. We and others have previously used similar methods to characterize acute intoxications with GHB [25,26], ecstasy [27], and methylphenidate [28].

## Results

The electronic search in the hospital data-base in a total of 172,207 ED patients during the study period identified 930 patients with notions of cocaine or related terms in the electronic record. Of these patients, 174 presented with acute medical problems related to cocaine use as verified by the full chart review. Of these 174 patients, 9 subjects were excluded because blood serum tests were negative for cocaine. Of the total 165 cases included in the present study, 151 (92%) reported cocaine abuse within 12 hours prior to presentation. Fourteen (8%) patients without self-reported cocaine use but with a positive blood serum test for cocaine and sympathetic toxicity were judged as being acutely intoxicated with cocaine. The demographic data are presented in Table 1. The annual number of presentations due to cocaine-related acute medical problems per total patients seen in the ED was 27/36,660 cases in 2007, 48/40,021 cases in 2008, 39/41,744 cases in 2009, 44/43,025 cases in 2010, and 7/10,757 cases from January to March 2011. The mean patient age ± SD was 32 ± 9 years and most were male and unemployed. Most patients were admitted to the ED at night and 98 (59%) patients were brought to the ED by ambulance. Intravenous or nasal administrations of cocaine were most common and equally frequent. Most patients used other drugs together with cocaine (Table 1). According to the blood serum tests, 25 (15%) patients abused cocaine alone whereas in 140 (85%) patients at least one additional substance was identified in the blood. Ethanol, opioids, cannabis, and benzodiazepines were the most commonly detected drugs while amphetamines were infrequently found (Table 1).

**Table 1:** Patient characteristics

	**Number of cases, N = 165 (%)**	
**Gender**		
Male	121 (73)	
Female	44 (27)	
**Age/years**		
16-20	15 (9)	
21-30	70 (42)	
31-40	54 (33)	
>40	26 (16)	
**Time of presentation**		
Night arrival (20:00 – 8:00 h)	106 (64)	
Weekend arrival (Friday 17:00 h – Monday 8:00 h)	70 (42)	
**Context of drug use**		
Recreational abuse	150 (91)	
Suicide attempt	7 (4)	
Unintentional/criminal	6 (4)	
Not reported	2 (1)	
**Route of cocaine administration**		
Intravenous	46 (28)	
Nasal	46 (28)	
Inhalational	4 (2)	
Oral	6 (4)	
Not reported	63 (38)	
**Concomitant drug use**	**Self-reported**	**Laboratory-confirmed**
None	32(19)	27 (16)
Ethanol	76 (46)	68 (41)
Opioids	43 (26)	63 (38)
Cannabis	29 (18)	59 (36)
Benzodiazepines	18 (11)	28 (17)
Amphetamines (including ecstasy)	6 (4)	2 (1)
GHB, GBL	4 (2)	1 (1)
LSD	1 (1)	0 (0)
**History of chronic cocaine use**		
Yes	102 (62)	
No	29 (18)	
Not reported	34 (21)	
**Employment**		
Yes	29 (18)	
No	107 (65)	
Not reported	29 (18)	

The clinical patient characteristics are summarized in Table 2. Among all 165 cases, 21 (13%) presented with severe intoxication but there were no fatalities. Severe intoxication was more frequent among polyintoxicated patients compared to intoxication with cocaine alone (Table 2). All 21 cases of severe toxicity and all 7 cases with a hospital stay longer than 24 hours were due to intoxication with cocaine and at least one other substance. The most commonly reported symptoms were chest pain, palpitations, anxiety, restlessness, somnolence, weakness, nausea/vomiting, and psychotic symptoms. Psychiatric symptoms were reported in 106 (64%) of the patients (Table 2). Hypertension and/or tachycardia were present in about half of the patients (Table 2). Two cases of myocardial infarction (one with and one without ST elevation) were observed in the multidrug user group. Both patients were admitted to the Intensive Care Unit and underwent coronary angiography. Decreased consciousness (GCS < 13) at any time during the ER presence was predominantly observed in the multidrug user group. Twenty-five (18%) of the 138 patients with multiple drug use had a GCS score below 13 any time during presence at the ED. Of these patients, all co-ingested either ethanol (10 cases) or opioids (11 cases) or both (4). Only 2 of the 27 patients in the cocaine alone using group had a GCS score below 13; one of these reported co-use of baclofen and the other co-use of GHB. Three cases of self-limited generalized seizures reportedly occurred in the total sample of 165 patients. Two of the three patients used heroin and one ethanol in addition to cocaine and one had a history of epilepsy. One patient with cocaine and barbiturate use suffered an ischemic stroke and was admitted to the neurology ward.

**Table 2:** Clinical characteristics of acute cocaine intoxication

	**All cases**	**Use of cocaine with other drugs**	**Use of cocaine alone**
	**No. (%)**	**No. (%)**	**No. (%)**
	**Total N = 165**	**Total N = 138**	**Total N = 27**
**Severity of poisoning**			
Severe poisoning	21 (13)	21 (15)	0 (0)*
**Cardiovascular**			
Chest pain	35 (21)	32 (23)	3 (11)
Palpitations	32 (19)	23 (17)	9 (33)
Dyspnea	21 (13)	16 (12)	5 (19)
Hypertension (systolic pressure >140 mmHg)	87 (53)	70 (51)	17 (63)
Tachycardia (>100 beats per minute)	72 (44)	62 (45)	10 (37)
Myocardial infarction	2 (1)	2 (1)	0 (0)
Hyperthermia >38.0°C	15 (9)	12 (9)	3 (11)
Hypoxemia (oxygen saturation <95%)	20 (12)	18 (13)	2 (7)
**Psychiatric**			
Anxiety	60 (36)	46 (33)	14 (52)
Restlessness	60 (36)	47 (34)	13 (48)
Hallucinations, paranoia, or psychosis	23 (14)	14 (10)	9 (33)
Agitation	37 (22)	28 (20)	9 (33)
Depression	32 (19)	28 (20)	4 (15)
Insomnia	18 (11)	18 (13)	0 (0)
Suicide ideation	15 (9)	12 (9)	3 (11)
Disorientation	17 (10)	15 (11)	2 (7)
**Neurologic**			
Impaired consciousness, GCS <9	17 (10)	15 (11)	2 (7)
Impaired consciousness, GCS 9-12	10 (6)	10 (7)	0 (0)
Impaired consciousness, GCS <13	27 (16)	25 (18)	2 (7)
Dizziness	24 (15)	21 (15)	3 (11)
Headache	16 (10)	14 (10)	2 (7)
Paresthesia	15 (9)	11 (8)	4 (15)
Seizure	3 (2)	2 (1)	1 (4)
Tremor	3 (2)	3 (2)	0 (0)
Stroke	1 (1)	1 (1)	0 (0)
**Miscellanous**			
Weakness	50 (30)	49 (36)	1 (4)
Nausea or vomiting	25 (15)	21 (15)	4 (15)
Myocloni or muscle cramps	9 (5)	7 (5)	2 (7)
Muscle pain	12 (7)	10 (7)	2 (7)
Abdominal pain	15 (9)	13 (9)	2 (7)
Elevated creatine kinase (>250 U/L)	73 (44)	62 (45)	11 (41)

*significant group difference (Fisher’s exact test, *p < 0.05).

Severity of intoxication was not significantly associated with the route of drug administration. However, severe intoxications occurred in 7 of 46 patients (15%) with intravenous drug use (cocaine or other drug) and in 5 of 57 patients (9%) with nasal, oral or inhalational drug administration. Hospitalization longer than 24 h was needed in 1 patient (2%) with injected drug use and in 4 (7%) of the patients using other routes of cocaine administration.

Therapeutic measures included the administration of intravenous fluids in 80 (48%) patients and of benzodiazepines for the treatment of cardiovascular toxicity and psychostimulation in 52 (32%) cases. Naloxone and flumazenil were administered in 14 (8%) and 2 (1%) patients, respectively. Forty patients (24%) were evaluated by a consultant psychiatrist on site. One hundred and twenty four (75%) patients were discharged home and 31 (19%) were referred to a psychiatric institution. Psychiatric hospitalization was needed in 7 of the 27 patients who used cocaine alone (26%) and in 24 of the 138 patients who used cocaine with other substances (17%). Similarly, psychiatric evaluation by consulting psychiatrists on the ER occurred nonsignificantly more frequently among cocaine only intoxications (9 cases, 33%) compared with polyintoxications (31 cases, 22%) (p = 0.22). Severity of intoxication did not significantly influence the frequency of psychiatric evaluations. A consultant was present in 37 of 107 mild to moderate intoxications (26%) and in 3 of 18 severe intoxications (14%). However, psychiatric hospitalization occurred only in mild to moderate intoxications (31 patients of 144 or 22%) while all 21 severe intoxications remained in general medical care (p < 0.05).

All patients with acute myocardial infarction were admitted to the ICU where coronary angiography was performed in both and stenting in the one with ST elevation MI. The one patient with an ischemic stroke was admitted to the neurology ward. Nineteen (12%) patients left the ED against medical advice. Among 68 patients exhibiting minor toxicity only one (1%) stayed for more than 24 h, among 76 patients with moderate toxicity three (4%) stayed for more than 24 h, and among the 21 patients with severe toxicity three (14%) stayed for more than 24 h. Of the 21 patients with severe intoxication 18 (86%) were discharged within 24 h.

## Discussion

In this study we described acute medical problems in patients with blood-test confirmed cocaine use. Most patients were men who chronically abused cocaine and had no employment. Of note, 53% of the patients were over 30 years old in line with increases in age of patients with cocaine-related health problems in other European countries [1]. The patients who abused more than one drug did so most commonly by combining cocaine with ethanol, opioids or cannabis, as reported by others [12,29]. We found that such polydrug use lead to significantly more severe intoxications compared with the use of cocaine alone. Co-use of ethanol was predominant in the present and in the previously studied series of cocaine intoxications [2]. Ethanol co-use with cocaine forms cocaethylene but also increases the plasma levels of cocaine, cocaine-induced subjective and also its cardiostimulant effects [30-32]. Ethanol may therefore increase the toxicity of cocaine [31,33] consistent with our findings. In accordance with data from the US [12], most visits occurred during night time. In our study, cocaine was mostly abused nasally or intravenously while predominantly intravenous and inhalational use was reported in the US [12,34] and mainly nasal administration was documented in Spain [2]. It is possible that intravenous drug use is overrepresented in our sample because it may lead to increased utilization of the ED due to more severe complications. Severe toxicity was more frequent with injection drug use compared to other routes of administration but this association was not statistically significant in the present study. In our study and in line with the work by others [2,12], co-use of other stimulants such as amphetamines or methylphenidate was hardly ever reported and amphetamines were only sporadically detected in the blood. In a recent analysis of exposures reported to the Swiss Toxicological Information Centre, co-use of amphetamines was noted in about 10% of the cocaine user [35]. Use of GHB was very rarely reported in our study whereas 15% of GHB users who presented to an ED in Zurich reported co-ingestion of cocaine [25]. In Spain, co-use of GHB in patients with cocaine intoxication was reported in only 6% of patients [2]. Use of methylphenidate was not reported in our study in accordance with an analysis of a small number of methylphenidate users presenting to the Basel ED [28] who did not co-use cocaine. In contrast, cocaine users with opioid substitution therapy in the Basel area indicate that they misuse methylphenidate orally, nasally, or intravenously as a substitute for cocaine [36]. Thus, methylphenidate co-use may have gone undetected because, contrary to other amphetamines, it was not detected by the blood screening test. Taken together, the data indicate that cocaine is not typically combined with other stimulants but rather co-used with sedating drugs including opioids, ethanol, or cannabis.

Chief complaints associated with acute cocaine toxicity were mainly neuropsychiatric or cardiovascular. The most commonly reported symptoms included anxiety, restlessness, agitation, chest pain and palpitations similar to previous studies [12,13]. Sixty-four percent of the patients presented with psychiatric symptoms similar to the 55-61% reported by recent previous studies [2,29] but higher than the 31-37% reported by earlier studies [12,37]. Loss of consciousness occurred in the multidrug user group, mainly due to concomitant use of ethanol or opioids. Hypertension and tachycardia were the main clinical signs regardless whether cocaine was used alone or in combination with other substances. Twenty-one percent of the patients reported chest pain. Percentages of patients reporting chest pain ranged from 11-39% in other case-series [2,12,29,37]. Two (1%) patients suffered from cocaine-related acute MI. MI similarly occurred in 1% of the patients presenting with cocaine toxicity and in 6% [12] of those with cocaine-associated chest pain in other studies [20,21]. Cardiovascular problems including myocardial infarction and arrhythmias are well-known medical complications of cocaine use [29,38,39]. Cocaine use has been observed in about 25% of 18–45 year-old patients with myocardial infarctions in [40]. Myocardial infarction may result from vasospasms [41] and excessive sympathetic activation. However, most cardiac deaths occur in patients with chronic cocaine use [42] and may involve structural cardiovascular disease including myocardial hypertrophy and microangiopathy [43-46]. In the presents study, both cases of cocaine-induced myocardial infarction occurred in chronic cocaine users. Stenting of significant coronary artery stenosis was performed in one while no coronary flow limitation was detected in the other. Similar to acute MI, cocaine use is an important risk factor for ischemic or hemorrhagic stroke in younger patients without classical cardiovascular risk factors [8,47,48]. In the present study, there was one patient with cocaine-induced ischemic stroke without other known risk factors except for tobacco smoking. Potential mechanisms of cocaine-induced ischemic or hemorrhagic stroke include arterial hypertension, vasospasm, disruption of cerebrovascular autoregulation, and enhanced platelet aggregation [8,41,49]. Other severe neurological complications in our study included 3 cases of self-limited seizures (2%). In all cases the patients had used other substances in addition to cocaine (heroin in 2 cases and ethanol in 1 case). Furthermore, one patient had a known history of seizures. Self-limited seizures were reported in 3-4% of patients in previous studies [12,50].

In our study, the management of patients included administration of intravenous fluids and benzodiazepines to control central and peripheral manifestations of sympathetic toxicity. The majority of patients including most patients with severe acute intoxications could be discharged home after less than 24 hours of observation in the ED as shown previously [12,34]. However, a significant number of patients needed psychiatric evaluation and referral.

This study has several limitations. As in all retrospective studies, there was some missing data and the initial patient histories and clinical data were not recorded in a standardized manner. In addition, it is possible that a few patients with positive drug tests for cocaine and use before 12 h prior to presentation were misclassified as acute intoxications. Similarly, acute co-use of cannabis was likely overestimated based on positive blood tests. In contrast, there was no screen for GHB in all patients and co-use of GHB may have gone undetected. As in all such studies, data from only one large ED may not be representative and reflect local drug using trends. Additionally, severe toxicity may have been overrepresented because we act as referral center for the Basel area including parts of France and Germany.

Our study has also several strengths. We had detailed patient and clinical data documentations in contrast to studies based on coded diagnoses or analyses of poison center data. In addition, the exposure to cocaine was confirmed in the blood for all patients and confirmative blood tests results for co-used ethanol and illicit drugs were also available for the majority of patients.

## Conclusion

Patients with acute cocaine intoxications presented with sympathomimetic toxicity or psychiatric disorders. Most patients co-used other drugs and polydrug use was a risk factor for more severe toxicity. Most patients could be discharged within 24 h. However, the proportion of patients needing further psychiatric evaluation and treatment was remarkable.

## Competing interests

The authors declare that they have no competing interests.

## Authors’ contributions

MEL designed the study. FE, EL and MBr extracted data. MBo, EL and MEL analyzed data and wrote the manuscript. All authors have read and approved the final manuscript.
